# Lysosomal trafficking regulator restricts intracellular growth of *Coxiella burnetii* by inhibiting the expansion of *Coxiella*-containing vacuole and upregulating *nos2* expression

**DOI:** 10.3389/fcimb.2023.1336600

**Published:** 2024-01-11

**Authors:** Weiqiang Wan, Shan Zhang, Mingliang Zhao, Xuan OuYang, Yonghui Yu, Xiaolu Xiong, Ning Zhao, Jun Jiao

**Affiliations:** ^1^ College of Life Sciences, Southwest Forestry University, Kunming, China; ^2^ State Key Laboratory of Pathogen and Biosecurity, Beijing Institute of Microbiology and Epidemiology, Beijing, China

**Keywords:** *Coxiella burnetii*, lysosomal trafficking regulator, *Coxiella*-containing vacuole, Dot/Icm type IV secretion system, inducible nitric oxide synthase

## Abstract

*Coxiella burnetii* is an obligate intracellular bacterium that causes Q fever, a zoonotic disease typically manifests as a severe flu-illness. After invading into the host cells, *C. burnetii* delivers effectors to regulate the vesicle trafficking and fusion events to form a large and mature *Coxiella*-containing vacuole (CCV), providing sufficient space and nutrition for its intracellular growth and proliferation. Lysosomal trafficking regulator (LYST) is a member of the Beige and Chediak-Higashi syndrome (BEACH) family, which regulates the transport of vesicles to lysosomes and regulates TLR signaling pathway, but the effect of LYST on *C. burnetii* infection is unclear. In this study, a series of experiments has been conducted to investigate the influence of LYST on intracellular growth of *C. burnetii*. Our results showed that *lyst* transcription was up-regulated in the host cells after *C. burnetii* infection, but there is no significant change in *lyst* expression level after infection with the Dot/Icm type IV secretion system (T4SS) mutant strain, while CCVs expansion and significantly increasing load of *C. burnetii* appeared in the host cells with a silenced *lyst* gene, suggesting LYST inhibits the intracellular proliferation of *C. burnetii* by reducing CCVs size. Then, the size of CCVs and the load of *C. burnetii* in the HeLa cells pretreated with E-64d were significantly decreased. In addition, the level of iNOS was decreased significantly in LYST knockout THP-1 cells, which was conducive to the intracellular replication of *C. burnetii*. This data is consistent with the phenotype of L-NMMA-treated THP-1 cells infected with *C. burnetii*. Our results revealed that the upregulation of *lyst* transcription after infection is due to effector secretion of *C. burnetii* and LYST inhibit the intracellular replication of *C. burnetii* by reducing the size of CCVs and inducing *nos2* expression.

## Introduction

*Coxiella burnetii*, an obligate intracellular Gram-negative bacterium, is the etiological agent of Q fever (Reimer, 1993; Qin et al., 2022). In nature, arthropods (including ticks and mites) and domestic ruminants (including cattle, sheep and goats) are the main hosts of *C. burnetii.* The main routes of *C. burnetii* infection are tick bites or inhalation of contaminated aerosols (Maurin and Raoult, 1999; Raoult et al., 2005). People infected by *C. burnetii* cause acute Q fever clinically characterized by high fever, headache, fatigue even acute pneumonia (Vinetz et al., 2017). In rare cases, persistent infection of *C. burnetii* in local tissues or organs causes chronic Q fever, leading to serious complications such as pneumonia, endocarditis, myocarditis, and osteomyelitis, and will be accompanied by a higher mortality rate (Stein and Raoult, 1995; Kazar, 2005; Eldin et al., 2017; Straily et al., 2017; Ghanem-Zoubi et al., 2021). Chronic Q fever is more difficult to treat than acute Q fever, and patients often require prolonged antibiotic treatment (van Roeden et al., 2018). Serological and molecular biological studies of *C. burnetii* have demonstrated that it is almost distributed around the world (Georgiev et al., 2013), and many studies have reported that Q fever outbreaks and epidemics have occurred in dozens of provinces, municipalities, and autonomous regions in China, including Chongqing, Sichuan, Yunnan, Tibet, Gansu, and Guangzhou (El-Mahallawy et al., 2015a; El-Mahallawy et al., 2015b; Huang et al., 2021).

*C. burnetii* invades host cells through the endocytosis pathway and forms phagosomes in the cells, and *C. burnetii* deliver effectors into the cytosol of host cells through the Dot/Icm type IV secretion system (T4SS) to regulate various host cell functions, such as apoptosis (Lührmann et al., 2010; Klingenbeck et al., 2013; Schäfer et al., 2017), autophagy (Valdivia et al., 2014; Mansilla Pareja et al., 2017), ubiquitination (Voth et al., 2011), metabolism and vesicle transport system (Kohler and Roy, 2015), which delivers *C. burnetii* to lysosome, ultimately forming a specialized membrane-bound compartment called *Coxiella*-containing vacuole (CCV) (Beare et al., 2011; Valdivia et al., 2014; Roy et al., 2019). With the homologous fusion of CCVs and the heterologous fusion between CCVs and vesicles such as autophagosomes, lysosomes and late endocytic vesicles, a mature and single huge CCV will gradually form to provide sufficient space and nutrition for the intracellular growth of *C. burnetii* (Kohler and Roy, 2015).

Lysosomal transport regulatory factor (LYST) is a highly conserved homologous protein in mammals, which is classified as a member of the Beige and Chediak-Higashi syndrome (BEACH) family due to a special BEACH region (Burgess et al., 2009; Cullinane et al., 2013). LYST regulates the transport of vesicles to lysosomes in host cells (Holland et al., 2014) and specifically controls interferon regulatory factors 3 (IRF3)/TIR domain-containing adapter-inducing interferon β (TRIF-β), inducing endosomal TLR signaling pathways (Westphal et al., 2017). Inhibition of LYST function results in abnormal phagosome maturation, leading to impaired formation of activation-induced rab7^+^ endosomal/phagosomal compartment, which is the major milestones in the TRIF signaling pathway (Westphal et al., 2017). LYST single gene mutation will lead to abnormal autophagy and defective vesicles transport, thus giving rise to phagosome enlargement (Cullinane et al., 2013; Sepulveda et al., 2015; Lattao et al., 2021).

In this study, we found that the expression of *lyst* was up-regulated in host cells after infection with *C. burnetii*, and the up-regulation of *lyst* expression was closely related to the secretion of *C. burnetii* effector. Further investigation showed that LYST inhibited the intracellular replication of *C. burnetii* by restricting the enlargement of CCVs and up-regulating the expression of *nos2*. The findings provide a novel clue for elucidating the pathogenic mechanism of *C. burnetii* and identify a novel potential therapeutic target for Q fever.

## Results

### Promotion of *C. burnetii* growth in host cells with knockdown of *lyst*

**Figure 1:**
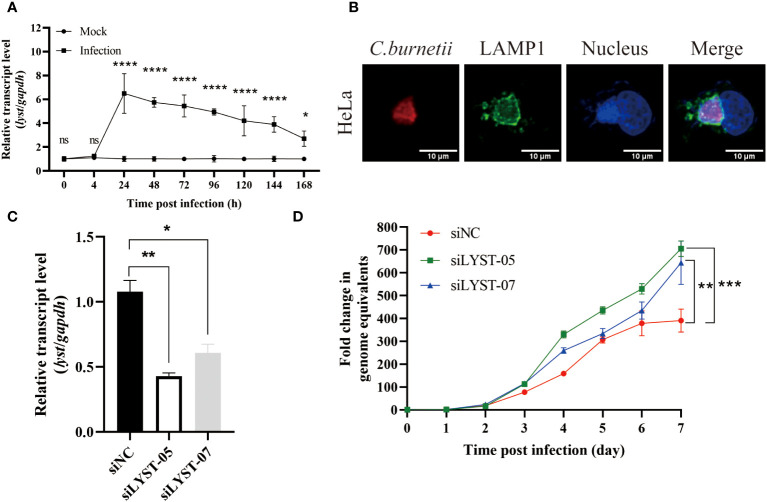
Phenotype of HeLa cells infected with *C*. *burnetii.* **(A)** The relative mRNA expression level of *lyst* in HeLa cells was analyzed after *C. burnetii* infection. Data are representative of three independent experiments and bars represent the mean ± SD. *, p<0.05, **, p<0.01, ***, p<0.001, ****, p<0.0001, ns, not significant; **(B)** CCVs (green) in HeLa cells were stained at 3 days post infection; **(C)** Inhibition efficiency of siRNA on *lyst* mRNA expression in HeLa cells. Data are representative of three independent experiments and bars represent the mean ± SD. *, p<0.05, **, p<0.01; **(D)** The growth curve of *C*. *burnetii* in HeLa cells. Data are representative of three independent experiments and bars represent the mean ± SD. **, p<0.01, ***, p<0.001 in comparison with control group at the day 7.

**Figure 2:**
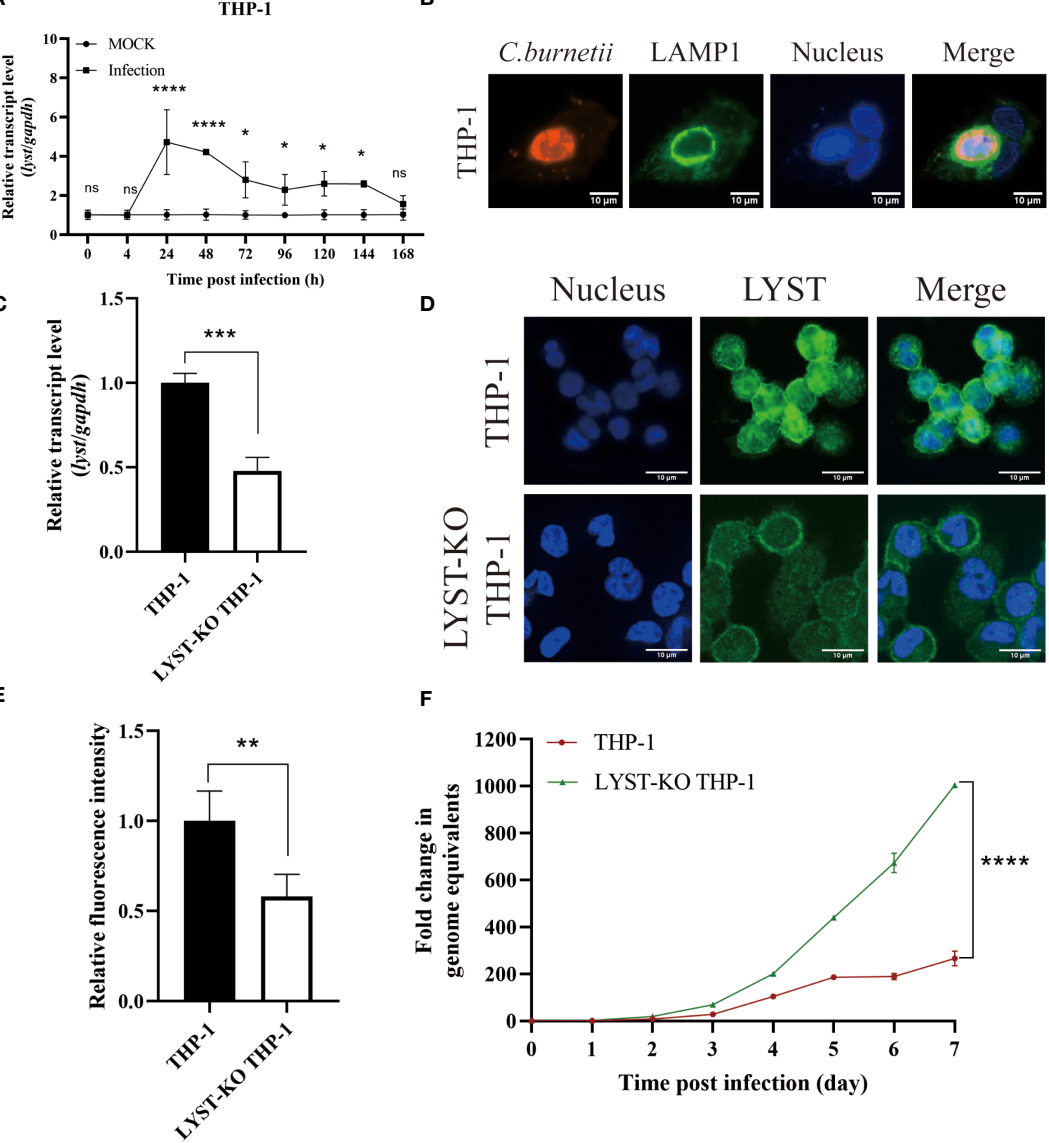
Phenotype of THP-1 cells in infected with *C*. *burnetii*. **(A)** The relative mRNA expression level of *lyst* in THP-1 cells was analyzed after *C. burnetii* infection. Data are representative of three independent experiments and bars represent the mean ± SD. *, p<0.05, ****, p<0.0001, ns, not significant; **(B)** CCVs (green) in THP-1 cells were stained at 3 days post infection; **(C)** Knockout efficiency of *lyst* mRNA expression in LYST-KO THP-1 cells. Data are representative of three independent experiments and bars represent the mean ± SD. ***, p<0.001; **(D)** LYST (green) was stained in THP-1 cells and LYST-KO THP-1 cells; **(E)** Relative fluorescence intensity of LYST in THP-1 cells and LYST-KO THP-1 cells. Data are representative of three independent experiments and bars represent the mean ± SD. **, p<0.01; **(F)** The growth curve of *C*. *burnetii* in THP-1 cells and LYST-KO THP-1 cells. Data are representative of three independent experiments and bars represent the mean ± SD. ****, p<0.0001 in comparison with control group at the day 7.

The transcription level of *lyst* mRNA in *C. burnetii* infected Hela cells was detected at different time points. As a result, it showed that the mRNA transcription levels of *lyst* were approximately 6-fold increased at 24 hours after infection, compared with uninfected controls (**Figure 1A**), indicating that *C. burnetii* infection caused up-regulation of mRNA expression of *lyst* in HeLa cells, and this result was also obtained in THP-1 cells (**Figure 2A**). At 72 hours after infection with *C. burnetii*, a large CCV that contained the lysosomal marker Lamp1 was observed in the host cells in fluorescence confocal analysis (**Figures 1B**, **2B**) (Roy et al., 2019).

To assess the impact of LYST on intracellular replication of *C. burnetii*, HeLa cells were transfected with siRNAs (siLYST-05 or siLYST-07) to silence the expression of *lyst*, achieving an inhibition efficiency of approximately 50% (**Figure 1C**). Subsequently, the *lyst*-silenced HeLa cells were infected with *C. burnetii*, and then *C. burnetii* load was detected. As a result, the intracellular replication level of *C. burnetii* was enhanced significantly in the *lyst*-silenced cells (**Figure 1D**).

THP-1 cells were notoriously difficult to transfect as almost all well-established transfection approaches (Lorkowski et al., 2014; Tang et al., 2018), so we used LYST-knockout THP-1 (purchased from Cyagen, China) to investigate the effect of LYST on the intracellular reproduction of *C. burnetii* in THP-1 cells. The knockout efficiency of LYST in THP-1 was about 50% by qRT-PCR and cellular immunofluorescence assay (**Figures 2C–E**). At the same time, *C. burnetii* were infected with the LYST-knockout THP-1 cells and the bacterial load of *C. burnetii* was significantly increased (**Figure 2F**).

In conclusion, the expression level of *lyst* was significantly up-regulated in both HeLa cells and THP-1 cells after *C. burnetii* infection, and *C. burnetii* load were significantly increased in the *lyst* gene knockdown/konckout host cells, suggesting that LYST inhibits intracellular replication of *C. burnetii*.

### The level of *lyst* expression correlates with the Dot/Icm type IV secretion system of *C. burnetii*

In the course of infection, a variety of effectors of *C. burnetii* with different functions were delivered through T4SS and interact with the functional regulatory molecules of host cells to promote the development of CCVs and enhance its intracellular replication.

**Figure 3:**
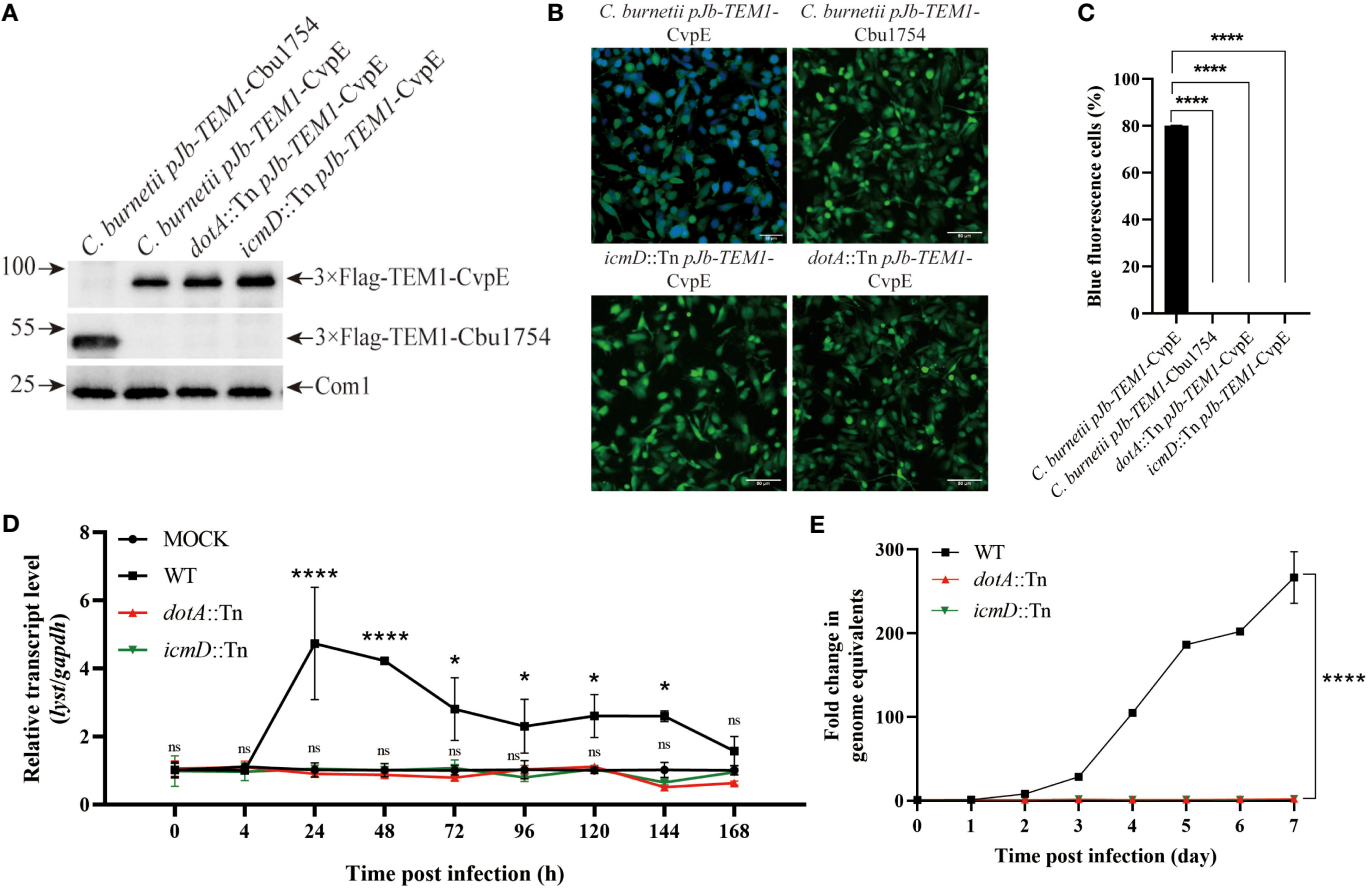
Identification of the up-regulation of *lyst* expression level in host cells was related to the secretion of *C*. *burnetii* effectors. **(A)** The fusion proteins expression of *C*. *burnetii pJb-TEM1*-CvpE, *C*. *burnetii pJb-TEM1*-Cbu1754, *dotA*::Tn *pJb-TEM1*-CvpE or *icmD*::Tn *pJb-TEM1*-CvpE cultured in ACCM-2 for 7 days were detected by Western blotting. **(B)** TEM translocation assays of *C*. *burnetii pJb-TEM1*-CvpE, *C*. *burnetii pJb-TEM1*-Cbu1754, *dotA*::Tn *pJb-TEM1*-CvpE or *icmD*::Tn *pJb-TEM1*-CvpE. **(C)** Proportion of blue fluorescence cells in each group. Data are representative of three independent experiments and bars represent the mean ± SD. ****, p<0.0001; **(D)** The relative mRNA expression level of *lyst* in THP-1 cells was analyzed after *dotA*::Tn, *icmD*::Tn or wile type *C. burnetii* infection. Data are representative of three independent experiments and bars represent the mean ± SD. *, p<0.05, ****, p<0.0001, ns, not significant; **(E)** The growth curve of *dotA*::Tn, *icmD*::Tn or wile type *C*. *burnetii* in THP-1 cells. Data are representative of three independent experiments and bars represent the mean ± SD. ****, p<0.0001 in comparison with control group at the day 7.

In order to explore the relationship between the up-regulation of *lyst* expression after infection and the secretion of *C. burnetii* effectors, THP-1 cells were differentiated and infected with *C. burnetii pJb-TEM1-*CvpE [positive control, a T4SS effector (Lührmann et al., 2010; Larson et al., 2015)], *C. burnetii pJb-TEM1-*Cbu1754 [negative control, a structural protein (Valdivia et al., 2014; Steiner et al., 2021)], *dotA*::Tn *pJb-TEM1-*CvpE or *icmD*::Tn *pJb-TEM1-*CvpE and subjected to TEM translocation assays according to the LiveBLAzer™ FRET—B/G Loading Kit. The results showed that *dotA*::Tn and *icmD*::Tn did not transport 3×Flag-TEM1-CvpE to the host cytoplasm (**Figures 3A–C**).

Then THP-1 cells were infected with wile type *C. burnetii*, *dotA*::Tn or *icmD*::Tn and the transcription level of *lyst* mRNA and intracellular replication level within 7 days were detected. The results showed that *dotA*::Tn and *icmD*::Tn infection had no effect on *lyst* expression (**Figure 3D**) and two strains hardly prolifed in host cells (**Figure 3E**), indicating that *C. burnetii* regulates *lyst* expression by effectors secretion, and affects CCVs development and intracellular replication of *C. burnetii*.

### LYST inhibits the intracellular proliferation of *C. burnetii* by reducing CCVs size

**Figure 4:**
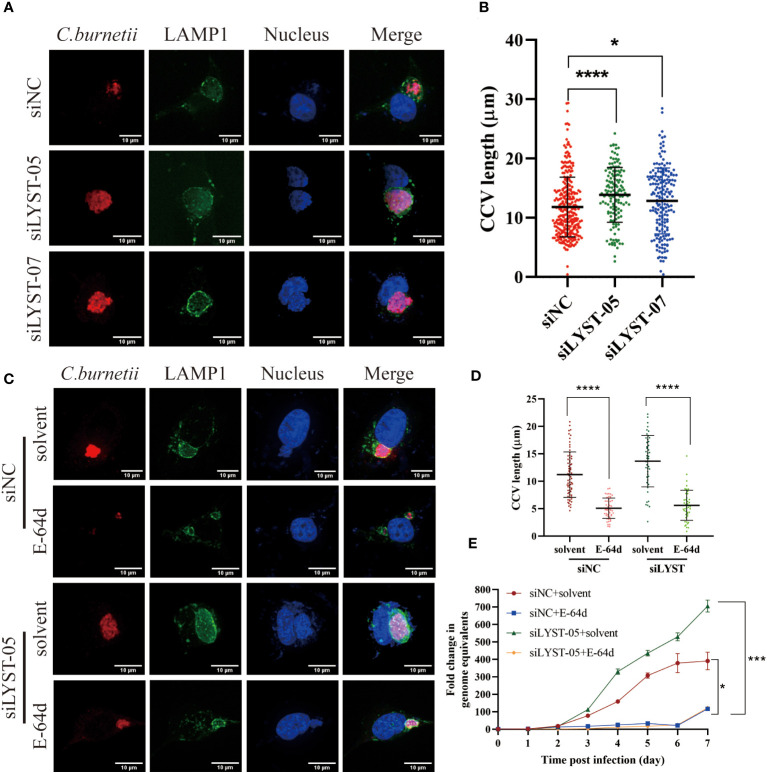
LYST inhibits the intracellular growth of *C*. *burnetii* by reducing CCVs expansion. **(A)** HeLa cells were transfected by different siRNAs, and CCVs (Green) were detected at 5 days post infection; **(B)** HeLa cells were transfected by different siRNAs, and CCV diameters were compared at 5 days post infection. Data are representative of two independent experiments and bars represent the mean ± SD. *, p<0.05, ****, p<0.0001.C. HeLa cells were transfected with siRNAs for 2 days and then pretreated with E-64d, following by infected with *C*. *burnetii*, CCVs (green) in HeLa cells were stained at 5 days post infection; **(D)** HeLa cells were transfected with siRNAs for 2 days and then pretreated with E-64d, CCV diameters were measured. Data are representative of two independent experiments and bars represent the mean ± SD. ****, p<0.0001; **(E)** HeLa cells were transfected with siRNAs for 2 days and then pretreated with E-64d, intracellular growth curves of *C*. *burnetii* were analyzed. Data are representative of three independent experiments and bars represent the mean ± SD. *, p<0.05, ***, p<0.001 in comparison with control group at the day 7.

With the infection of *C. burnetii* infection, a mature and huge CCV will gradually form in the host cell, which provides an acidic environment (pH~5.0) and adequate nutrients for the intracellular growth of *C. burnetii* (Kohler and Roy, 2015). After *C. burnetii* infection, both the CCVs size and the *C. burnetii* loads were significantly increased in the *lyst-*silenced cells compared with the unsilenced cells (**Figures 4A, B**), suggesting that LYST inhibits the intracellular proliferation of *C. burnetii* by reducing CCVs size in host cells.

Aloxistatin (E-64d), a cysteine proteases inhibitor, has been shown to inhibit the enlargement of cellular lysosomes (Tanabe et al., 2000; Huynh et al., 2004; Yamada et al., 2005) to restrict the intracellular proliferation of pathogenic microorganisms (Beverley et al., 2008). To investigate whether LYST hinders the intracellular replication of *C. burnetii* by suppressing CCVs expansion, HeLa cells were treated with E-64d and then infected with *C. burnetii*. The result showed that both the CCVs size and the intracellular *C. burnetii* load were significantly reduced in E-64d treated cells compared with the E-64d untreated cells (**Figures 4C, D**). These findings indicate that E-64d effectively inhibited the intracellular proliferation of *C. burnetii* by suppressing CCVs expansion. Furthermore, when *lyst*-silenced HeLa cells were pretreated with E-64d, both the CCVs size (**Figures 4C, D**) and the *C. burnetii* load (**Figure 4E**) were significantly reduced, also indicating that LYST plays a role in inhibiting intracellular proliferation of *C. burnetii* through reducing the size of CCVs.

### Regulation of *nos2* expression by LYST affects intracellular proliferation of *C. burnetii*

Inducible nitric oxide synthase (iNOS) is encoded by nitric oxide synthase 2 gene (*nos2*) in macrophages. It can be induced and activated by many compounds such as bacterial lipopolysaccharide (LPS), type I/II interferon, and cytokines to stimulate macrophages to release nitric oxide (NO), a powerful bactericide (Zamboni and Rabinovitch, 2004; Hill et al., 2011), which effectively inhibits the proliferation of intracellular bacteria (Howe et al., 2002; Matthiesen et al., 2023). This process is one of the important bactericidal mechanisms of macrophages (Kleinert et al., 2004; Tailleux et al., 2011; Schairer et al., 2014; Bogdan, 2015). The use of nitric oxide synthase inhibitor (N^G^-Monomethyl-L-arginine, L-NMMA) effectively inhibits the activity of iNOS (Chakravortty et al., 2002; Brennan et al., 2004).

**Figure 5:**
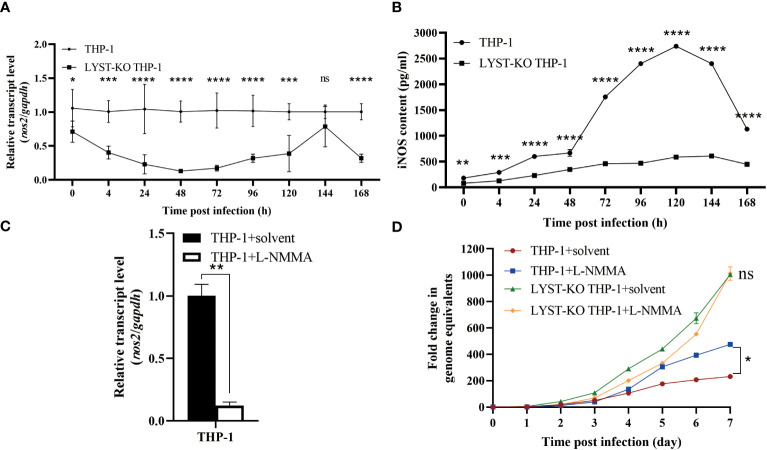
LYST inhibits the intracellular reproduction of *C*. *burnetii* by upregulating *nos2* expression in host cells. **(A)** The expression level of *nos2* in THP-1 cells after infection with *C. burnetii*. Data are representative of three independent experiments and bars represent the mean ± SD. *, p<0.05, **, p<0.01, ***, p<0.001, ****, p<0.0001, ns, not significant; **(B)** The iNOS content was measured in THP-1 cells after *C*. *burnetii* infection. Data are representative of three independent experiments and bars represent the mean ± SD. *, p<0.05, **, p<0.01, ***, p<0.001, ****, p<0.0001; **(C)** Relative expression level of *nos2* in THP-1 cells pretreated with L-NMMA. Data are representative of three independent experiments and bars represent the mean ± SD. **, p<0.01; **(D)** Intracellular growth curves of *C. burnetii* in THP-1 cells pretreated with L-NMMA. Data are representative of three independent experiments and bars represent the mean ± SD. *, p<0.05, ***, p<0.001, ns, not significant in comparison with control group at the day 7.

To investigate the potential impact of LYST on the intracellular proliferation of *C. burnetii* by activating the expression of *nos2*, the LYST-knockout THP-1 cells were induced to adhere in advance and subsequently infected with *C. burnetii*. The total RNA samples extracted from the infected cells at different time points were used to detect the expression of *nos2* mRNA in the host cells, and the supernatants of cell culture were quantitatively detected iNOS by ELISA. The results showed that a significant decrease both in *nos2* mRNA level and iNOS content in LYST-knockdown THP-1 cells infected with *C. burnetii* compared with those of the infected normal THP-1 cells (**Figures 5A, B**).

Then the THP-1 cells were pretreated with L-NMMA to suppress *nos2* expression with about 88% inhibition efficiency (**Figure 5C**). After infection with *C. burnetii*, the L-NMMA-treated THP-1 cells were significantly enhanced in intracellular *C. burnetii* load compared to that of controls (**Figure 5D**), suggesting that the expression of *nos2* inhibits the intracellular proliferation of *C. burnetii*. However, there was no significant difference in intracellular *C. burnetii* load between LYST-knockdown THP-1 cells pretreated with L-NMMA and untreated LYST-knockdown THP-1 cells (**Figure 5D**), suggesting that LYST inhibits the reproduction of *C. burnetii* by inducing *nos2* expression in host cells.

## Discussion

*C. burnetii* is an obligate intracellular Gram-negative bacterium. After infecting host cells, it will be phagocytosed to form a phagosome which developed into CCV to provide a large and suitable environment and abundant nutrients for the growth and reproduction of *C. burnetii* in the cytosol of host cells (Christie et al., 2011; Kohler and Roy, 2015). Therefore, the development and maturation of CCV is a necessary for *C. burnetii* to multiply in host cells and sustain stable infection (Best and Abu Kwaik, 2019).

LYST, a member of the BEACH family (Cullinane et al., 2013), is a transporter protein that regulates the transport of small vesicles to lysosomes in host cells (Holland et al., 2014). Loss of functional LYST results in the abnormal maturation of phagosomes and enlargement of lysosomal associated organelles (Barbosa et al., 1996; Kaplan et al., 2008). Studies have shown that *lyst* gene-deficient mice exhibited increased susceptibility to bacteria (Westphal et al., 2017). Furthermore, study showed that host cells could inhibit intracellular growth of *Leishmania amazonensis* by suppressing the development of parasitic vesicles through LYST (Beverley et al., 2008). This process may be an innate immune response of host cells against intracellular pathogens. However, whether LYST affects the replication of *C. burnetii* in host cells remains unclear currently. This study aims to conduct an initial investigation into the influence of LYST on the intracellular reproduction of *C. burnetii*.

In our study, it was found that after infection with *C. burnetii*, the mRNA expression level of *lyst* in host cells was up-regulated, while both the CCVs size and *C. burnetii* load in the host cells silenced with *lyst* were increased significantly compared with those of the normal cell control. Therefore, it is extrapolated that LYST might inhibit the intracellular proliferation of *C. burnetii* by suppressing CCVs expansion after infection. This conjecture was confirmed by the experiment in the host cells pretreated with E-64d, a drug that inhibit the expansion of vesicles in cells, and the host cells pretreated with E-64d showed smaller CCVs and lower bacterial loads after infection with *C. burnetii*.

Since LYST can specifically affect the TLR3/TRIF-mediated endosomal TLR signal transduction pathway (Westphal et al., 2017), and the upregulation of *nos2* is partly dependent on TLR3/TRIF (Zhang et al., 2016), we used RT-qPCR and ELISA to respectively detect the mRNA expression level of *nos2* and iNOS content in LYST-knockout THP-1 cells infected with *C. burnetii* at different times. It was found that the mRNA expression level of *nos2* and the content of iNOS in LYST-knockout THP-1 cells were significantly lower than those in normal THP-1 cells controls, while the loads of *C. burnetii* was significantly enhanced in LYST-knockout THP-1 cells and the THP-1 cells pretreated with a competitive inhibitor of nitric oxide synthase (L-NMMA).

## Conclusion

In summary, our study found that the transcription of *lyst*, a gene previously shown to regulate lysosome size, was up-regulated in host cells after infection with *C. burnetii*, which were confirmed by experiments in the present study, demonstrating that the expression of *lyst* in host cells after infection is related to the effector secretion of *C. burnetii* and LYST not only inhibits the expansion of CCVs, thereby reducing the space for the growth and reproduction of *C. burnetii*, but also results in up-regulation of *nos2* expression by activating the innate immune response of macrophages to catalyze the production of nitric oxide (NO), a key defense molecule. By the combination of the two pathways, the growth and reproduction of *C. burnetii* could be effectively inhibited in macrophages. Therefore, the results in the present study lay a theoretic basis for developing novel drugs against *C. burnetii* and providing novel therapeutic strategies for Q fever.

## Materials and methods

### Bacterial strains and cell culture

The *C. burnetii* Nine Mile RSA439, phase II strain, clone 4 (NMII) was preserved in our laboratory and propagated in freshly prepared modified acidified citrate cysteine medium (ACCM-2) at 37°C with 5% CO_2_ and 2.5% O_2_.

HeLa cells were purchased from ATCC and cultured in DMEM containing 10% fetal bovine serum (FBS) at 37°C with 5% CO_2_. THP-1 cells were purchased from ATCC and grown in RPMI 1640 medium with 10% FBS and 0.1% 2-mercaptoethanol at 37°C with 5% CO_2_.

### Cell processing and immunofluorescence staining of the CCVs membrane and LYST

Before the experiment, Hela cells or THP-1 cells were seeded in a 12-well cell culture plate at the required cell density. Aloxistatin (E-64d, MCE, E8640) was added to monolayer cells at 1 μM for 4 hours prior to infection. N^G^-Monomethyl-L-arginine (L-NMMA, MCE, M7033) was added to monolayer cells at 50 μM for 4 hours prior to infection. Cells were washed three times with sterile PBS and then infected with *C. burnetii* at a multiplicity of infection (MOI) of 2.

For staining the CCVs, cells were treated as follow at 5 dpi: 1) cells were fixed with 4% paraformaldehyde for 15 minutes; 2) cells were permeabilized with 0.2% TritonX-100 for 15 minutes; 3) incubated with 1% BSA at room temperature for 30 minutes to block the cells; 4) anti-*C. burnetii* serum (prepared in our laboratory) and goat anti-mouse IgG Alexa Flour 594 antibody (Abcam, ab150116), anti-LAMP-1 antibody (CTS, 9091S) and goat anti-rabbit IgG Alexa Flour 488 antibody (Abcam, ab150113) were incubated successively, and each antibody was incubated at room temperature for 1 hour; 5) After washing with PBS, the cell nuclei were stained with DAPI at room temperature for 3 to 5 minutes. After each staining step, wash 3 times with sterile PBS for 5 minutes each time; 6) the coverslips were removed and mounted on slides with Prolong Gold antifade mounting solution (Thermo, p36935) to observe CCVs, and the results were photographed and saved.

For staining the LYST, THP-1 cells and LYST-KO THP-1 cells were differentiated with PMA (200 nM) for 48 h, 1) the cells were fixed, permeabilized and block (as show above); 2) anti-LYST antibody (Abcam, ab220481) and goat anti-rabbit IgG Alexa Flour 488 antibody (Abcam, ab150113) were incubated successively, and each antibody was incubated at room temperature for 1 hour; 3) follow-up operations are as described previously; 4) the relative fluorescence intensity of 488nm was calculated.

### Electroporation of *C. burnetii* with plasmids

The *pJb-TEM1*-CvpE and *pJb-TEM1*-Cbu1754 were introduced into electrocompetent wild type *C. burnetii* respectively, and additionally, *pJb-TEM1*-CvpE was introduced into electrocompetent two T4SS mutant stains (*dotA*::Tn and *icmD*::Tn) at 1800 V, 500 Ω, 25 µF and duration between 9-13 msec. Immediately, 900 µl of RPMI 1640 medium was added into the mixture at room temperature (Bonazzi et al., 2015). Subsequently, the electroporated bacteria was inoculated into 5 ml of ACCM-2 containing 1% FBS and cultured at 37 °C, 2.5% O_2_, 5% CO_2_ for 7 days. Finally, 100 µl of the culture is coated with ACCM-2 plates containing kanamycin (375 µg/ml) and 0.2% agarose to isolate individual *C. burnetii pJb-TEM1-*CvpE, *C. burnetii pJb-TEM1-*Cbu1754, *dotA*::Tn *pJb-TEM1-*CvpE and *icmD*::Tn *pJb-TEM1-*CvpE.

### TEM translocation assays

THP-1 cells were differentiated into adherent, macrophage- like cells and infected with *C. burnetii* strains carrying β-lactamase fusions at a MOI of 100. 6X CCF4-AM Substrate Loading Solution to cells to 1X final concentration at 1 day post infection and incubate the plate at room temperature for 60–120 minutes. Samples were observed under a fluorescence microscope and detection of the blue coumarin emission (~450 nm) and green fluorescein emission (~520 nm), and positive cells were marked blue.

### Gene silencing and efficiency detection

HeLa cells were seeded in the cell culture plate at a density of 1×105 cells/ml in advance. After the cells adhere to the well, two lyst-specific siRNA (siLYST-05 and siLYST-04, **Table 1**) were transfected into HeLa cells with Lipofectamine RNAiMAX Reagent for 2 days. The total cellular RNA was extracted and reverse-transcribed into cDNA by TransScript one-step method. Subsequently, the cDNA was analyzed for lyst transcription levels by RT-qPCR, with the required primers listed in **Table 2**. The expression level was determined relative to the internal reference gene (gapdh).

**Table 1:** Sequences of siRNA for *lyst* gene silence.

siRNA	Sequence (5’-3’)
siLYST-05	GACGUUACCUUGAAUCAUA
siLYST-07	GAACCAGUGAUUAGACUUA
*siNC	UUCUCCGAACGUGUCACGUTT

*Negative Control: non-specific siRNA.

**Table 2:** Primers for RT-qPCR.

Primer	Sequence (5’-3’)
h*lyst*-R	GCACATCAAGTTTGGCTTTACT
h*lyst*-F	TACTCGAAAACCTCACTCAAGG
*nos2*-R	GAATGTGCTGTTTGCCTCGG
*nos2*-F	GATGGGAGAAGGGGATGAGC
h*gapdh*-R	GGAAGATGGTGATGGGATT
h*gapdh*-F	AACGGATTTGGTCGTATTG

### Detection of intracellular growth of *C. burnetii* and cell *nos2* expression level

The cells were infected with *C. burnetii* at a MOI of 2, and then the unabsorbed *C. burnetii* was washed away with sterile PBS 4 hours later. Cell samples were collected every 24 hours to extract DNA, and DNA samples were detected by qPCR to determine the bacterial loads of *C. burnetii* (primers such as **Table 3**).

**Table 3:** Primers and probes for qPCR.

Primer	Sequence (5’-3’)
com1-F	AAAACCTCCGCGTTGTCTTCA
com1-R	GCTAATGATACTTTGGCAGCGTATTG
com1-probe	5’6-FAM-AGAACTGCCCATTTTTGGCGGCCA-3’-BHQ1

According to the above infection method, THP-1 cells were infected with C. burnetii. Total RNA was extracted at different time points after infection, and the relative expression level of nos2 was detected by RT-qPCR (required primers such as **Table 2**). At the same time, the supernatant of cell culture was collected and the content of iNOS was detected by Human iNOS ELISA KIT.

## Data availability statement

The original contributions presented in the study are included in the article/supplementary material. Further inquiries can be directed to the corresponding author.

## Author contributions

WW: Investigation, Methodology, Writing – original draft. SZ: Investigation, Writing – original draft. MZ: Formal analysis, Writing – original draft. XO: Formal analysis, Writing – original draft. YY: Formal analysis, Writing – original draft. XX: Formal analysis, Writing – review & editing. NZ: Writing – review & editing. JJ: Funding acquisition, Project administration, Writing – review & editing.
